# Supplementary material for: Decoding Thalamic Glial Interplay in Multiple Sclerosis Through Proton Magnetic Resonance Spectroscopy and Positron Emission Tomography
Source: Int J Mol Sci. 2025 Sep 5;26(17):8656. doi: 10.3390/ijms26178656 (PMC12428814; doi:10.3390/ijms26178656)
Supplement: Supplementary file 1 [file ijms-26-08656-s001.zip › ijms-3805975-supplementary.pdf]

## Supplementary Methods

*MSFC Protocol*

The Expanded Disability Status Scale (EDSS) [1] evaluates physical disability with a strong emphasis on ambulation [1]. The 25-foot timed walk (25FTW) test assesses lower leg function and walking speed [2]. The 9-hole peg test (9HPT) assesses arm and hand function [3]. The Paced Auditory Serial Addition Test (PASAT) assesses cognitive function [4].

The average of two trials for the 25FTW and 9HPT (dominant hand) was calculated. The scores of PASAT, 9HPT and 25FTW acquired in this study were converted to z-scores as previously described [5] following MSFC guidelines. The formula for z-score transformation is shown below. The average of these three scores was used to estimate the composite MSFC score. For z-score transformations, the reference MS population was derived from the NMSS TASK Force-pooled dataset, as recommended in the guidelines [2, 6].

$$\text{MSFC score} = [(1/\ \textit{9-HPT dominant arm}) - 0.0439) / 0.0101 + [ - (\text{Average 25-Foot Walk} - 9.5353) / 11.4058] + (\text{PASAT} - 45.0311) / 12.0771]/ 3.0 \quad \text{(S1)}$$

It is important to note that the sign of z-scores of the T25W test was multiplied by -1 after the z-score transformation, as recommended in the guidelines [2, 6]. This adjustment ensures that the direction of change in the z-scores for all three components is uniform, meaning that a decrease in the z-score indicates deterioration [2, 5].

*$^1$H-MRS*

The acquisition and processing techniques for $^1$H-MRS have been described in detail previously [7-9]. Briefly, a single voxel $^1$H spectrum was acquired using a modified sLASER sequence with VAPOR water suppression (TR/TE=5000/30, 64 water-suppressed averages, 4 embedded unsuppressed water references, a spectral width of 6 kHz). B0 shimming was performed using FAST(EST)MAP [7, 10]. The unsuppressed embedded water spectra were used for both spectral quantification and residual eddy current correction [9]. Each free induction decay was individually saved for processing, including eddy current, frequency, and phase corrections. After eddy current correction, frequency and phase corrections were performed automatically using the MRspa software (https://www.cmrr.umn.edu/downloads/mrspa) in MATLAB before summing the spectra for LCModel (version 6.3–0G) (http://s-provencher.com/lcmodel.shtml) quantitation.

To assess the percentage of tissue fractions within the $^1$H-MRS voxel of interest, tissue classification images were first created using a standard brain atlas (Mayo Clinic Adult Lifespan Template, MCALT, www.nitrc.org/projects/mcalt) [11]. An MRS mask was then created using the T1-weighted MPRAGE volume and the MRS DICOM files and applied to the tissue classification images which allowed quantification of percentage of tissue fractions for gray matter (GM), white matter (WM), and cerebrospinal fluid (CSF) (see average values in supplementary **Table S1**).

*$^1$H-MRS Data Quantification*

Quantification of the pre-processed sLASER spectra was conducted using LCModel 6.3 (Stephen Provencher, Oakville, Ontario, Canada). LCModel is a fitting routine that utilizes a comprehensive library of model spectra for various metabolites [12]. The LCModel

basis set contained model spectra for macromolecules and 19 metabolites: This library includes spectra for alanine, aspartate, ascorbate, glycerophosphocholine (GPC), phosphocholine (PC), creatine (Cr), phosphocreatine (PCr), $\gamma$-aminobutyric acid, glucose, glutamine (Gln), glutamate (Glu), glutathione (GSH), myo-inositol (mIns), lactate, N-acetylaspartate (NAA), N-acetylaspartylglutamate (NAAG), phosphoethanolamine (PE), scyllo-inositol, taurine, and experimentally obtained macromolecules [13].

$^{1}$H-MRS metabolite levels were calculated in two ways: first, as estimated metabolite concentrations (millimolar, mM) using tissue water as an internal reference, and second, by normalizing the estimated concentrations of other metabolites to tCr, expressed as metabolite/tCr ratios. Estimated metabolite concentrations were calculated by correcting LCModel metabolite values using tissue-specific water content and individual tissue volume fractions for GM, WM, and CSF, as described in more detail below [14]. The T2 relaxation time of tissue water obtained in the thalamus (96 ms) [15] was taken into account in LCModel fitting for concentration (mM) calculations. Corrections for T1 and T2 relaxation of metabolites were not applied, as we expected metabolite concentrations to be relatively insensitive to changes in T1 and T2 relaxation times due to the high TR and low TE used in this study.

With respect to the correlation coefficient r values between metabolites (estimated via LCModel), Cr and PCr, as well as GPC and PCho, had high cross-correlations (absolute r >0.7) at 3T, so only the sums were reported. This criteria were applied based on recent recommendations of the $^{1}$H-MRS expert's consensus initiative [16].. NAAG was not reliably quantified in this study. The following metabolites were reliably quantified: Glu, Gln, NAA, tCho, mIns, and tCr.

Metabolite concentrations (mM) were determined for each participant after correcting for tissue water content and cerebrospinal fluid contributions in the $^{1}$H-MRS voxel using the water-scaling option in LCModel as descried earlier [17, 18]. This method is based on water content in the tissue, which varies based on the WM, GM, and CSF fractions (fGM, fWM, and fCSF) in the $^{1}$H-MRS voxel. Metabolite concentrations in CSF are negligible except for lactate and glucose. This leads to partial volume effects that must be addressed for accurate measurements. Voxel water concentration was calculated assuming water content (wc) of 82% for GM, 72% for WM, and 100 % for CSF [9, 19, 20].

The GM, WM and CSF fractions within the $^{1}$H-MRS voxel were estimated for each participant. Thus, the LCModel output was corrected for tissue fraction containing water. For partial volume correction, tissue fraction containing water was divided by 1-fCSF which is equal to sum of fGM and fWM as follows [21]:

$$\text{Tissue fraction containing water: } (\text{fGM} * \text{wc\_gm}) + (\text{fWM} * \text{wc\_wm}) + (\text{fCSF} * \text{wc\_csf}). \quad \text{(S2)}$$

W_gm, w_wm and w_csf are the water content in GM, WM and CSF, respectively.

The following formula was used to correct the LCModel output data:

$$\text{Cmet} = \text{LCModel\_output} * \frac{\text{Rw}}{\text{Rm}} \text{x} \frac{[(\text{fGM} * \text{wc\_gm}) \ + (\text{fWM} * \text{wc\_wm}) + (\text{fCSF} * \text{wc\_csf})]}{(\text{fGM} + \text{fWM})} \quad \text{(S3)}$$

$R_{H20}$= Attenuation factor of water signal for given repetition and echo times and tissue specific T2.

T2 tissue H20= 96 ms for thalamus.[15]

$$\text{Rw} = e^{-\text{TE}/\text{T2\_tissue\_H20}} \quad \text{(S4)}$$

fGM: Gray matter fraction in the thalamus MRS voxel measured for each particiapant.

fWM: White matter fraction in the thalamus MRS voxel measured for each particiapant.

fCSF: Cerebral spinal fluid fraction in the thalamus MRS voxel measured for each particiapant.

Rm: Attenuation factor of metabolites = 1 (default value in LCModel).

LCModel recommends ignoring T1 relaxation at least for TR≥4000ms since T1 is approximately the same for water and the metabolites, and the two corrections then partially cancel. LCModel recommends ignoring T2 relaxation correction of metabolites if TE is equal to or smaller than 30 ms [9]. In other words, if the T2 relaxation time differs between metabolites, the corresponding changes in ratios of the signals for metabolites and water (NAA/water) which are caused by signal attenuation are small.

*$^{11.}$ C-ER176 TSPO PET*

Each participant's own T1-weighted MRI was registered to PET images for GM, WM and atlas segmentation. Analysis revealed no significant differences in $^{11}$C-ER176 PET SUVR between pwMS with and without lesions in the left or right cerebellar crus (p=0.35 and p=0.34). As a result, the cerebellar crus was chosen as the reference region to compute standard uptake value ratios. Previous research has demonstrated that the low-affinity TSPO binding genotype is relatively rare (approximately 10%) [22], and $^{11}$C-ER176 binding reliably detects signal across all genotypes, including high-, mixed-, and low-affinity binding types [23-26]. Therefore, no additional correction was performed for the low-affinity TSPO binding genotype

## Supplementary Discussion

The estimated metabolite concentrations (mM) provide direct quantification and eliminate the need to assume tCr stability. However, estimating metabolite concentrations from spectra involves several assumptions, which may introduce bias due to variations in tissue water concentration (e.g., due to edema or atrophy) and differences in relaxation times. In our analysis, we observed a systemic increase in NAA, Glu, Gln, tCr, and mI concentrations in pwMS compared to controls (supplementary **Table S2**). This elevation may reflect a combination of methodological biases and/or pathological effects on metabolite concentration estimates, highlighting the complexity of interpreting these findings in the context of the disease. Gracien et al. (2016) reported that an increase in T1 relaxation times of the cerebral cortex and deep gray matter structures, including the thalamus, in pwMS than in controls [27]. Specifically, the study observed an increase in tissue T1 relaxation time in thalamus in pwMS. Although we did not measure thalamic T1 relaxation times in pwMS and controls, such increases in T1 relaxation times in the thalamus of pwMS, if present, would decrease metabolite concentrations. These results suggest that the systemic increase in estimated metabolite concentrations observed in our study is unlikely to be due to elevated T1 relaxation times in thalamic tissue. Srinivasan et al. (2005) reported no significant alterations in the T2 relaxation times of metabolites (NAA, Cho, and Cr) between pwMS and controls [28]. Based on this, we assumed in our study that T2 relaxation times of metabolites remained unchanged. Furthermore, Srinivasan et al. (2005) demonstrated a decrease in $^{1}$H-MRS metabolite T1 relaxation times [28]. If a similar decrease in T1 relaxation times occurred in pwMS, it could have resulted in an underestimation of metabolite concentrations. Thus, these findings collectively suggest that the potential bias observed in our study is unlikely to be influenced by changes in metabolite T1 or T2 relaxation times.

Estimated metabolite concentrations (mM) are typically calculated using tissue water concentration as an internal reference, assuming that it remains relatively stable within the region of interest. However, edema or atrophy may cause variations in tissue water concentrations, potentially introducing biases. Edema may elevate water concentration in pwMS, which, if present within the thalamic $^{1}$H-MRS voxel, would likely decrease

estimated metabolite concentrations rather than cause the observed increase. However, atrophy reduces tissue water concentration, which may lead to an increase in estimated metabolite concentrations. In MS, thalamic innate immune activity may contribute to both edema and atrophy, with a predominant effect depending on the disease stage and severity. While we did not measure thalamic tissue water concentration, a previous study reported that thalamic tissue water remains unchanged between pwMS and controls [29]. Nevertheless, we performed partial volume correction to reduce to bias due to alterations in tissue composition (GM, WM, CSF).

On $^1$H-MRS, both mIns (mM) (p=0.001) and Glu (mM) (p=0.028) estimated concentrations (mM) were increased in pwMS compared to controls after adjusting for age (supplementary **Table S2**). Notably, the observed increase in mIns (mM) is consistent with the results obtained using the mIns/tCr ratio. The observed elevation in estimated thalamic Glu concentrations (mM) in pwMS, contrasted with unchanged Glu/tCr ratios. This discrepancy may arise from two complementary factors. First, a subtle (albeit non-significant) elevation in tCr (mean: 7.42 mM in pwMS vs. 6.89 mM in controls; p = 0.16) could stabilize the Glu/tCr ratio, masking disease-specific Glu alterations when normalized. Second, estimated metabolite concentrations may carry inherent biases from tissue water or relaxation time assumptions, which metabolite ratios (e.g., Glu/tCr) inherently minimize. These findings underscore the value of reporting both water-referenced concentrations and metabolite ratios to disentangle metabolic dynamics, as each metric captures distinct aspects of thalamic pathology in MS.

We observed a positive correlation with tCho (mM), tCr (mM) and EDSS severity (supplementary **Table S6)**. The correlation of absolute tCho (mM)—but not tCho/tCr—with EDSS suggests that the tCho/tCr ratio may be influenced by subtle changes in tCr levels in pwMS.

In conclusion, both estimated metabolite concentrations and metabolite/tCr ratios captured key aspects of MS pathology, particularly the relationship between mIns and normalized thalamic volume, with both metrics (ratio to tCr and estimated concentrations) showing significant negative correlations (supplementary **Figure S1**, and **Tables S4 and S5)**. Overall, these findings highlight mIns as a robust marker of thalamic pathology and innate immune reaction. However, potential biases in estimated concentrations, such as those related to tissue water content or relaxation times, necessitate cautious interpretation.

## References


1. Kurtzke JF. Rating neurologic impairment in multiple sclerosis: an expanded disability status scale (EDSS). Neurology. 1983;33:1444-52. doi:10.1212/wnl.33.11.1444.
2. Cutter GR, Baier ML, Rudick RA, Cookfair DL, Fischer JS, Petkau J, et al. Development of a multiple sclerosis functional composite as a clinical trial outcome measure. Brain. 1999;122 ( Pt 5):871-82. doi:10.1093/brain/122.5.871.
3. Goodkin DE, Hertsgaard D, Seminary J. Upper extremity function in multiple sclerosis: improving assessment sensitivity with box-and-block and nine-hole peg tests. Arch Phys Med Rehabil. 1988;69:850-4.
4. Gronwall DM. Paced auditory serial-addition task: a measure of recovery from concussion. Percept Mot Skills. 1977;44:367-73. doi:10.2466/pms.1977.44.2.367.
5. Fischer JS, Rudick RA, Cutter GR, Reingold SC. The Multiple Sclerosis Functional Composite measure (MSFC): an integrated approach to MS clinical outcome assessment. Multiple Sclerosis Journal. 1999;5:244-50. doi:10.1177/135245859900500409.
6. Fischer JS, Jak A, Kniker J, Rudick R, Cutter G. Multiple Sclerosis Functional Composite (MSFC): administration and scoring manual. New York: National Multiple Sclerosis Society. 2001.
7. Oz G, Tkáč I. Short-echo, single-shot, full-intensity proton magnetic resonance spectroscopy for neurochemical profiling at 4 T: validation in the cerebellum and brainstem. Magnetic resonance in medicine. 2011;65:901-10. doi:10.1002/mrm.22708.
8. Tkac I, Starcuk Z, Choi IY, Gruetter R. In vivo 1H NMR spectroscopy of rat brain at 1 ms echo time. Magn Reson Med. 1999;41:649-56.

9. Deelchand DK, Adanyeguh IM, Emir UE, Nguyen T-M, Valabregue R, Henry P-G, et al. Two-site reproducibility of cerebellar and brainstem neurochemical profiles with short-echo, single-voxel MRS at 3T. Magnetic Resonance in Medicine. 2015;73:1718-25. doi:https://doi.org/10.1002/mrm.25295.
10. Gruetter R, Tkáč I. Field mapping without reference scan using asymmetric echo-planar techniques. Magnetic Resonance in Medicine. 2000;43:319-23. doi:https://doi.org/10.1002/(SICI)1522-2594(200002)43:2<319::AID-MRM22>3.0.CO;2-1.
11. Schwarz CG, Gunter JL, Ward CP, Kantarci K, Senjem ML, Petersen RC, et al. P3-382: METHODS TO IMPROVE SPM12 TISSUE SEGMENTATIONS OF OLDER ADULT BRAINS. Alzheimer's & Dementia. 2018;14:P1240-P1. doi:https://doi.org/10.1016/j.jalz.2018.06.1744.
12. Provencher SW. Automatic quantitation of localized in vivo H-1 spectra with LCModel. Nmr in Biomedicine. 2001;14:260-4. doi:10.1002/nbm.698.
13. Deelchand DK, Henry P-G, Uğurbil K, Marjańska M. Measurement of transverse relaxation times of J-coupled metabolites in the human visual cortex at 4 T. Magnetic resonance in medicine. 2012;67:891-7. doi:10.1002/mrm.23080.
14. Muhlert N, Atzori M, De Vita E, Thomas DL, Samson RS, Wheeler-Kingshott CAM, et al. Memory in multiple sclerosis is linked to glutamate concentration in grey matter regions. Journal of Neurology, Neurosurgery & Psychiatry. 2014;85:833-9. doi:10.1136/jnnp-2013-306662.
15. Sedlacik J, Boelmans K, Löbel U, Holst B, Siemonsen S, Fiehler J. Reversible, irreversible and effective transverse relaxation rates in normal aging brain at 3T. NeuroImage. 2014;84:1032-41. doi:https://doi.org/10.1016/j.neuroimage.2013.08.051.
16. Near J, Harris AD, Juchem C, Kreis R, Marjańska M, Öz G, et al. Preprocessing, analysis and quantification in single-voxel magnetic resonance spectroscopy: experts' consensus recommendations. NMR in Biomedicine. 2021;34:e4257. doi:https://doi.org/10.1002/nbm.4257.
17. Quadrelli S, Mountford C, Ramadan S. Hitchhiker's Guide to Voxel Segmentation for Partial Volume Correction of In Vivo Magnetic Resonance Spectroscopy. Magn Reson Insights. 2016;9:1-8. doi:10.4137/mri.S32903.
18. Near J, Harris AD, Juchem C, Kreis R, Marjańska M, Öz G, et al. Preprocessing, analysis and quantification in single-voxel magnetic resonance spectroscopy: experts' consensus recommendations. NMR Biomed. 2021;34:e4257. doi:10.1002/nbm.4257.
19. Randall LO. CHEMICAL TOPOGRAPHY OF THE BRAIN. Journal of Biological Chemistry. 1938;124:481-8. doi:https://doi.org/10.1016/S0021-9258(18)74053-2.
20. Norton WT, Poduslo SE, Suzuki K. Subacute Sclerosing Leukoencephalitis*†‡: II. Chemical Studies Including Abnormal Myelin and an Abnormal Ganglioside Pattern. Journal of Neuropathology & Experimental Neurology. 1966;25:582-97. doi:10.1097/00005072-196610000-00006.
21. Bednařík P, Henry PG, Khowaja A, Rubin N, Kumar A, Deelchand D, et al. Hippocampal Neurochemical Profile and Glucose Transport Kinetics in Patients With Type 1 Diabetes. J Clin Endocrinol Metab. 2020;105:479-91. doi:10.1210/clinem/dgz062.
22. Kreisl WC, Fujita M, Fujimura Y, Kimura N, Jenko KJ, Kannan P, et al. Comparison of [(11)C]-(R)-PK 11195 and [(11)C]PBR28, two radioligands for translocator protein (18 kDa) in human and monkey: Implications for positron emission tomographic imaging of this inflammation biomarker. Neuroimage. 2010;49:2924-32. doi:10.1016/j.neuroimage.2009.11.056.
23. Ikawa M, Lohith TG, Shrestha S, Telu S, Zoghbi SS, Castellano S, et al. 11C-ER176, a Radioligand for 18-kDa Translocator Protein, Has Adequate Sensitivity to Robustly Image All Three Affinity Genotypes in Human Brain. J Nucl Med. 2017;58:320-5. doi:10.2967/jnumed.116.178996.
24. Kreisl WC, Kim MJ, Coughlin JM, Henter ID, Owen DR, Innis RB. PET imaging of neuroinflammation in neurological disorders. Lancet Neurol. 2020;19:940-50. doi:10.1016/S1474-4422(20)30346-X.
25. Viviano M, Barresi E, Simeon FG, Costa B, Taliani S, Da Settimo F, et al. Essential Principles and Recent Progress in the Development of TSPO PET Ligands for Neuroinflammation Imaging. Curr Med Chem. 2022;29:4862-90. doi:10.2174/0929867329666220329204054.
26. Cumbers GA, Harvey-Latham ED, Kassiou M, Werry EL, Danon JJ. Emerging TSPO-PET Radiotracers for Imaging Neuroinflammation: A Critical Analysis. Semin Nucl Med. 2024. doi:10.1053/j.semnuclmed.2024.09.007.
27. Gracien RM, Jurcoane A, Wagner M, Reitz SC, Mayer C, Volz S, et al. The Relationship between Gray Matter Quantitative MRI and Disability in Secondary Progressive Multiple Sclerosis. PLoS One. 2016;11:e0161036. doi:10.1371/journal.pone.0161036.
28. Srinivasan R, Sailasuta N, Hurd R, Nelson S, Pelletier D. Evidence of elevated glutamate in multiple sclerosis using magnetic resonance spectroscopy at 3 T. Brain. 2005;128:1016-25. doi:10.1093/brain/awh467.
29. Wylezinska M, Cifelli A, Jezzard P, Palace J, Alecci M, Matthews PM. Thalamic neurodegeneration in relapsing-remitting multiple sclerosis. Neurology. 2003;60:1949-54. doi:10.1212/01.wnl.0000069464.22267.95.

**Table S1.** Mean Values of GM, WM and CSF Fractions in Thalamic [1]H-MRS voxel in Patients with Multiple Sclerosis (pwMS) and Controls.

| Group | GM Fraction (Mean ± SD) | WM Fraction (Mean ± SD) | CSF Fraction (Mean ± SD) |
|---|---|---|---|
| Control | 40.65 ± 6.23 | 50.61 ± 8.50 | 8.73 ± 3.69 |
| pwMS | 35.61 ± 5.89 | 51.66 ± 8.49 | 12.72 ± 4.60 |

GM: Gray matter fraction over 100.; WM: White matter fraction over 100; CSF: Cerebral Spinal Fluid fraction over 100; SD: Standard deviation.

**Table S2.** [1]H MRS Concentrations (mM) of Controls and Patients with MS.

| | **Control (N=30)** | **MS (N=21)** | ***p*-Value (Age-Adjusted)** |
|---|---|---|---|
| [1]H-MRS metabolites (mM) | | | |
| tCho | 2.07 [1.96, 2.21] / 2.08 (± 0.22) | 2.07 [1.98, 2.39] / 2.19 (± 0.36) | 0.497 |
| NAA | 8.42 [7.94, 9.12] / 8.51 (± 0.73) | 9.11 [8.22, 9.51] / 9.02 (± 0.86) | 0.094 |
| Glu | 7.88 [7.06, 8.49] / 7.83 (± 1.12) | 8.67 [8.16, 9.49] / 8.72 (± 1.11) | **0.028** |
| Gln | 2.86 [2.47, 3.15] / 2.85 (± 0.73) | 3.50 [2.92, 3.81] / 3.37 (± 0.71) | 0.131 |
| mIns | 5.71 [5.57, 6] / 5.84 (± 0.64) | 7.12 [6.04, 8.16] / 7.25 (± 1.51) | **0.001** |
| tCr | 6.89 [6.44, 7.11] / 6.89 (± 0.62) | 7.27 [6.78, 7.79] / 7.42 (± 0.95) | 0.156 |

Data are shown as n (%) or median [IQR: 1st to 3rd quartile range] / mean (±SD). The variables were adjusted for "age at imaging" using linear regression model with "age at imaging" and "group: MS and control" as independent variables. The p-values reported are the p-values of the estimates for the "group" variable, indicating whether the group differences are statistically significant after adjusting for "age at imaging". tCr, total creatine; tCho, total choline; NAA, N-acetylaspartate; Glu, glutamate; Gln, glutamine; mIns, myo-inositol. P-values are rounded to three decimal places.

**Table S3.** Linear Regression Models Investigating Associations Between Age * Group (two levels: patients with MS and controls) Interaction with Imaging Variables.

**(a) Thalamus volume/TIV**

| | **Model With Interaction** | | | | **Model Without Interaction** | | | |
|---|---|---|---|---|---|---|---|---|
| *Variable* | *Estimates* | *std. Error* | *95% CI* | *p-Value* | *Estimates* | *std. Error* | *95% CI* | *p-Value* |
| Group:MS | -0.377 | 0.543 | -1.469 – 0.715 | 0.491 | -0.715 | 0.145 | -1.006 – -0.423 | **<0.001** |
| Age | 0.007 | 0.008 | -0.010 – 0.023 | 0.412 | 0.003 | 0.006 | -0.009 – 0.015 | 0.595 |
| Group x Age | -0.008 | 0.012 | -0.032 – 0.016 | 0.521 | | | | |
| Observations | 51 | | | | 51 | | | |
| $R^2$ / $R^2$ adjusted | 0.35 / 0.31 | | | | 0.35 0.32 | | | |

**(b) tCho/tCr**

| | **Model With Interaction** | | | | **Model Without Interaction** | | | |
|---|---|---|---|---|---|---|---|---|
| *Variable* | *Estimates* | *std. Error* | *95% CI* | *p-Value* | *Estimates* | *std. Error* | *95% CI* | *p-Value* |

| | | | | | | | | |
|---|---|---|---|---|---|---|---|---|
| Group:MS | -0.002 | 0.028 | -0.058 – 0.054 | 0.949 | -0.009 | 0.007 | -0.024 – 0.005 | 0.205 |
| Age | 0.000 | 0.000 | -0.001 – 0.001 | 0.909 | -0.000 | 0.000 | -0.001 – 0.001 | 0.916 |
| Group x Age | -0.000 | 0.001 | -0.001 – 0.001 | 0.774 | | | | |
| Observations | 51 | | | | 51 | | | |
| $R^2$ / $R^2$ adjusted | 0.04 / -0.02 | | | | 0.04 / -0.00 | | | |

**(c) tCho (mM)**

| | Model With Interaction | | | | Model Without Interaction | | | |
|---|---|---|---|---|---|---|---|---|
| *Variable* | *Estimates* | *std. Error* | *95% CI* | *p-Value* | *Estimates* | *std. Error* | *95% CI* | *p-Value* |
| Group:MS | -0.085 | 0.320 | -0.728 – 0.559 | 0.792 | 0.059 | 0.086 | -0.114 – 0.232 | 0.497 |
| Age | 0.004 | 0.005 | -0.006 – 0.014 | 0.405 | 0.006 | 0.004 | -0.001 – 0.013 | 0.117 |
| Group x Age | 0.003 | 0.007 | -0.011 – 0.018 | 0.643 | | | | |
| Observations | 50 | | | | 50 | | | |
| $R^2$ / $R^2$ adjusted | 0.09 / 0.03 | | | | 0.09 0.05 | | | |

**(d) NAA/tCr**

| | Model With Interaction | | | | Model Without Interaction | | | |
|---|---|---|---|---|---|---|---|---|
| *Variable* | *Estimates* | *std. Error* | *95% CI* | *p-Value* | *Estimates* | *std. Error* | *95% CI* | *p-Value* |
| Group:MS | -0.017 | 0.090 | -0.198 – 0.165 | 0.854 | -0.001 | 0.024 | -0.050 – 0.047 | 0.954 |
| Age | -0.002 | 0.001 | -0.005 – 0.000 | 0.091 | -0.002 | 0.001 | -0.004 – -0.000 | **0.034** |
| Group x Age | 0.000 | 0.002 | -0.004 – 0.004 | 0.861 | | | | |
| Observations | 51 | | | | 51 | | | |
| $R^2$ / $R^2$ adjusted | 0.10 / 0.05 | | | | 0.10 / 0.07 | | | |

**(e) NAA (mM)**

| | Model With Interaction | | | | Model Without Interaction | | | |
|---|---|---|---|---|---|---|---|---|
| *Variable* | *Estimates* | *std. Error* | *95% CI* | *p-Value* | *Estimates* | *std. Error* | *95% CI* | *p-Value* |
| Group:MS | -0.452 | 0.892 | -2.248 – 1.343 | 0.614 | 0.413 | 0.242 | -0.073 – 0.899 | 0.094 |
| Age | 0.002 | 0.014 | -0.026 – 0.029 | 0.911 | 0.011 | 0.010 | -0.009 – 0.031 | 0.280 |
| Group x Age | 0.020 | 0.020 | -0.020 – 0.060 | 0.319 | | | | |
| Observations | 50 | | | | 50 | | | |

| | | | | | | | | |
|---|---|---|---|---|---|---|---|---|
| $R^2$ / $R^2$ adjusted | 0.14 / 0.08 | | | | 0.12 / 0.08 | | | |

**(f) Glu/tCr**

| | Model With Interaction | | | | Model Without Interaction | | | |
|---|---|---|---|---|---|---|---|---|
| *Variable* | *Estimates* | *std. Error* | *95% CI* | *p-Value* | *Estimates* | *std. Error* | *95% CI* | *p-Value* |
| Group: MS | -0.026 | 0.135 | -0.298 – 0.246 | 0.848 | 0.047 | 0.036 | -0.025 – 0.120 | 0.195 |
| Age | -0.002 | 0.002 | -0.006 – 0.002 | 0.285 | -0.001 | 0.001 | -0.004 – 0.002 | 0.345 |
| Group x Age | 0.002 | 0.003 | -0.004 – 0.008 | 0.575 | | | | |
| Observations | 51 | | | | 51 | | | |
| $R^2$ / $R^2$ adjusted | 0.05 / -0.01 | | | | 0.04 / 0.00 | | | |

**(g) Glu (mM)**

| | Model With Interaction | | | | Model Without Interaction | | | |
|---|---|---|---|---|---|---|---|---|
| *Variable* | *Estimates* | *std. Error* | *95% CI* | *p-Value* | *Estimates* | *std. Error* | *95% CI* | *p-Value* |
| Group: MS | -0.573 | 1.263 | -3.115 – 1.969 | 0.652 | 0.776 | 0.343 | 0.085 – 1.466 | **0.028** |
| Age | -0.002 | 0.019 | -0.041 – 0.037 | 0.913 | 0.012 | 0.014 | -0.016 – 0.041 | 0.382 |
| Group x Age | 0.031 | 0.028 | -0.025 – 0.088 | 0.273 | | | | |
| Observations | 50 | | | | 50 | | | |
| $R^2$ / $R^2$ adjusted | 0.17 / 0.12 | | | | 0.15 / 0.12 | | | |

**(h) Gln/tCr**

| | Model With Interaction | | | | Model Without Interaction | | | |
|---|---|---|---|---|---|---|---|---|
| *Variable* | *Estimates* | *std. Error* | *95% CI* | *p-Value* | *Estimates* | *std. Error* | *95% CI* | *p-Value* |
| Group: MS | -0.093 | 0.097 | -0.287 – 0.102 | 0.343 | 0.018 | 0.026 | -0.034 – 0.071 | 0.484 |

| | | | | | | | | |
|---|---|---|---|---|---|---|---|---|
| Age | 0.001 | 0.001 | -0.002 – 0.004 | 0.553 | 0.002 | 0.001 | -0.000 – 0.004 | 0.067 |
| Group x Age | 0.003 | 0.002 | -0.002 – 0.007 | 0.240 | | | | |
| Observations | 51 | | | | 51 | | | |
| $R^2$ / $R^2$ adjusted | 0.13 / 0.07 | | | | 0.10 / 0.07 | | | |

**(i) Gln (mM)**

| | Model With Interaction | | | | Model Without Interaction | | | |
|---|---|---|---|---|---|---|---|---|
| *Variable* | *Estimates* | *std. Error* | *95% CI* | *p-Value* | *Estimates* | *std. Error* | *95% CI* | *p-Value* |
| Group: MS | -0.847 | 0.764 | -2.385 – 0.691 | 0.273 | 0.324 | 0.210 | -0.100 – 0.747 | 0.131 |
| Age | 0.010 | 0.012 | -0.014 – 0.033 | 0.402 | 0.022 | 0.009 | 0.005 – 0.040 | **0.012** |
| Group x Age | 0.027 | 0.017 | -0.007 – 0.061 | 0.118 | | | | |
| Observations | 50 | | | | 50 | | | |
| $R^2$ / $R^2$ adjusted | 0.27 / 0.22 | | | | 0.23 / 0.19 | | | |

**(j) mIns/tCr**

| | Model With Interaction | | | | Model Without Interaction | | | |
|---|---|---|---|---|---|---|---|---|
| *Variable* | Estimates | std. Error | 95% CI | *p-Value* | Estimates | std. Error | 95% CI | *p-Value* |
| Group: MS | 0.072 | 0.101 | -0.132 – 0.275 | 0.481 | 0.100 | 0.027 | 0.046 – 0.154 | **0.001** |
| Age | 0.002 | 0.001 | -0.001 – 0.005 | 0.165 | 0.002 | 0.001 | 0.000 – 0.005 | **0.034** |
| Groupx Age | 0.001 | 0.002 | -0.004 – 0.005 | 0.778 | | | | |
| Observations | 51 | | | | 51 | | | |
| $R^2$ / $R^2$ adjusted | 0.36 / 0.32 | | | | 0.36 0.33 | | | |

**(k) mIns (mM)**

| | Model With Interaction | | | | Model Without Interaction | | | |
|---|---|---|---|---|---|---|---|---|
| *Variable* | *Estimates* | *std. Error* | *95% CI* | *p-Value* | *Estimates* | *std. Error* | *95% CI* | *p-Value* |
| Group:MS | 0.314 | 1.150 | -2.000 – 2.628 | 0.786 | 1.068 | 0.310 | 0.444 – 1.691 | **0.001** |
| Age | 0.030 | 0.017 | -0.005 – 0.066 | 0.088 | 0.039 | 0.013 | 0.013 – 0.064 | **0.004** |
| Group x Age | 0.017 | 0.026 | -0.034 – 0.069 | 0.499 | | | | |
| Observations | 50 | | | | 50 | | | |
| $R^2$ / $R^2$ adjusted | 0.42 / 0.38 | | | | 0.41 / 0.39 | | | |

**(l) [11]C-ER176 PET SUVR**

| | Model With Interaction | | | | Model Without Interaction | | | |
|---|---|---|---|---|---|---|---|---|
| *Variable* | Estimates | std. Error | 95% CI | *p-Value* | Estimates | std. Error | 95% CI | *p-Value* |
| Group:MS | 0.068 | 0.018 | 0.032 – 0.104 | **<0.001** | 0.071 | 0.018 | 0.035 – 0.108 | **<0.001** |
| Age | -0.001 | 0.001 | -0.003 – 0.001 | 0.270 | 0.000 | 0.001 | -0.001 – 0.002 | 0.978 |
| Group x Age | 0.002 | 0.001 | -0.000 – 0.005 | 0.098 | | | | |
| Observations | 50 | | | | 50 | | | |
| $R^2$ / $R^2$ adjusted | 0.31 / 0.27 | | | | 0.27 / 0.24 | | | |

The tables present the effects of Age * Group (patient with MS and controls) interaction on imaging variables (dependent variables). When the interaction was not significant, the model run without the interaction term, while keeping the age in the model (model without interaction). In the model without interaction term, the estimate for Group:MS represents the average difference in the dependent variable between the patient with MS and controls controlling for age. A positive estimate suggests that the mean value of the dependent variable is higher in the pwMS compared to controls, after adjusting for age. Bold p-values denote statistical significance ($p < 0.05$). Name of the dependent imaging variables are indicated at the top of each table. Thalamus volume/Total intracranial volume (TIV) ratio was scaled by multiplying 1000. P-values are rounded to three decimal places. P-values below 0.001 are reported as <0.001. Abbreviations: tCr, total creatine; tCho, total choline; NAA, N-acetylaspartate; Glu, glutamate; Gln, glutamine; mIns, myo-inositol.

**Table S4.** Partial Pearson Correlation (age-adjusted) Between Thalamic [1]H-MRS metabolite concentration (mM) and [11]C-ER176 PET SUVR.

| | MS | | | Control | | |
|---|---|---|---|---|---|---|
| **[1]H-MRS metabolites** | **r** | **95% CI** | ***p*-value** | **r** | **95% CI** | ***p*-value** |

| | | | | | | |
|---|---|---|---|---|---|---|
| tCho (mM) | 0.36 | [-0.06, 0.78] | 0.118 | -0.26 | [-0.63, 0.11] | 0.199 |
| NAA (mM) | 0.38 | [-0.04, 0.8] | 0.094 | -0.53 | [-0.86, -0.2] | **0.005** |
| Glu (mM) | 0.23 | [-0.21, 0.67] | 0.337 | -0.34 | [-0.7, 0.02] | 0.08 |
| Gln (mM) | 0.24 | [-0.2, 0.68] | 0.316 | -0.21 | [-0.59, 0.17] | 0.299 |
| mIns (mM) | 0.47 | [0.07, 0.87] | **0.036** | -0.06 | [-0.44, 0.32] | 0.771 |
| tCr (mM) | 0.32 | [-0.11, 0.75] | 0.174 | -0.25 | [-0.62, 0.12] | 0.202 |

See **Table 2** for abbreviations of metabolites: r, correlation coefficient; MS (number of participants =21); Control (number of participants = 28); Bold p-values denote statistical significance ($p < 0.05$). The associations were assessed using age-adjusted Pearson correlation. P-values are rounded to three decimal places. Abbreviations: tCr, total creatine; tCho, total choline; NAA, N-acetylaspartate; Glu, glutamate; Gln, glutamine; mIns, myo-inositol.

**Table S5.** Partial Pearson Correlation (age-adjusted) Between Thalamic $^{1}$H-MRS Metabolite Concentration (mM) and Normalized Thalamus Volume.

| | MS | | | Control | | |
|---|---|---|---|---|---|---|
| **$^{1}$H-MRS metabolites** | **r** | **95% CI** | ***p*-value** | **r** | **95% CI** | ***p*-value** |
| tCho (mM) | -0.54 | [-0.92, -0.16] | **0.015** | -0.1 | [-0.48, 0.28] | 0.626 |
| NAA (mM) | -0.28 | [-0.71, 0.15] | 0.226 | -0.12 | [-0.49, 0.25] | 0.547 |
| Glu (mM) | -0.21 | [-0.65, 0.23] | 0.386 | -0.11 | [-0.48, 0.26] | 0.574 |
| Gln (mM) | -0.28 | [-0.71, 0.15] | 0.234 | 0.09 | [-0.29, 0.47] | 0.635 |
| mIns (mM) | -0.59 | [-0.95, -0.23] | **0.006** | -0.18 | [-0.55, 0.19] | 0.364 |
| tCr (mM) | -0.37 | [-0.79, 0.05] | 0.113 | -0.01 | [-0.39, 0.37] | 0.953 |

See Table 2 for abbreviations of metabolites: r, correlation coefficient; MS (number of participants =21); Control (number of participants = 29); Bold p-values denote statistical significance ($p < 0.05$). The associations were assessed using age-adjusted Pearson correlation. P-values are rounded to three decimal places. Abbreviations: tCr, total creatine; tCho, total choline; NAA, N-acetylaspartate; Glu, glutamate; Gln, glutamine; mIns, myo-inositol.

***Table S6.*** Correlation of $^{1}$H-MRS metabolites (mM) with Clinical Metrics in Patients with MS.

| | MSFC (N=20) | | | PASAT z-score (N=20) | | | EDSS (N=20) | | |
|---|---|---|---|---|---|---|---|---|---|
| **$^{1}$H-MRS metabolites** | **Rho** | **95% CI** | **P-value** | **Rho** | **95% CI** | **P-value** | **Rho** | **95% CI** | **P-value** |
| tCho (mM) | -0.26 | [-0.71, 0.19] | 0.285 | 0.15 | [-0.31, 0.61] | 0.537 | 0.48 | [0.09, 0.87] | **0.033** |
| NAA (mM) | -0.37 | [-0.8, 0.06] | 0.120 | 0.07 | [-0.39, 0.53] | 0.780 | 0.39 | [-0.02, 0.8] | 0.093 |
| Glu (mM) | -0.2 | [-0.65, 0.25] | 0.401 | 0.19 | [-0.26, 0.64] | 0.432 | 0.36 | [-0.06, 0.78] | 0.116 |
| Gln (mM) | -0.12 | [-0.58, 0.34] | 0.632 | 0.15 | [-0.31, 0.61] | 0.536 | 0.32 | [-0.11, 0.75] | 0.171 |
| mIns (mM) | -0.35 | [-0.78, 0.08] | 0.136 | -0.28 | [-0.72, 0.16] | 0.238 | 0.33 | [-0.09, 0.75] | 0.152 |
| tCr (mM) | -0.42 | [-0.84, 0.00] | 0.076 | 0.09 | [-0.37, 0.55] | 0.709 | 0.50 | [0.11, 0.89] | **0.023** |

Age-adjusted Partial Spearman correlation coefficient (rho) is presented with confidence interval [95% CI]. This non-parametric method is appropriate given the non-normal distribution of some of these clinical disability measures. Bold p-values indicate statistically significant associations ($p < 0.05$). P-values are rounded to three decimal places. Abbreviations: tCr, total creatine; tCho, total choline; NAA, N-acetylaspartate; Glu, glutamate; Gln, glutamine; mIns, myo-inositol; PASAT, Paced Auditory Serial Addition Test; 9-HPT, 9-Hole Peg Test from dominant hand; 25FTW, Timed 25-Foot Walk Test; MSFC, Multiple Sclerosis Functional Composite.

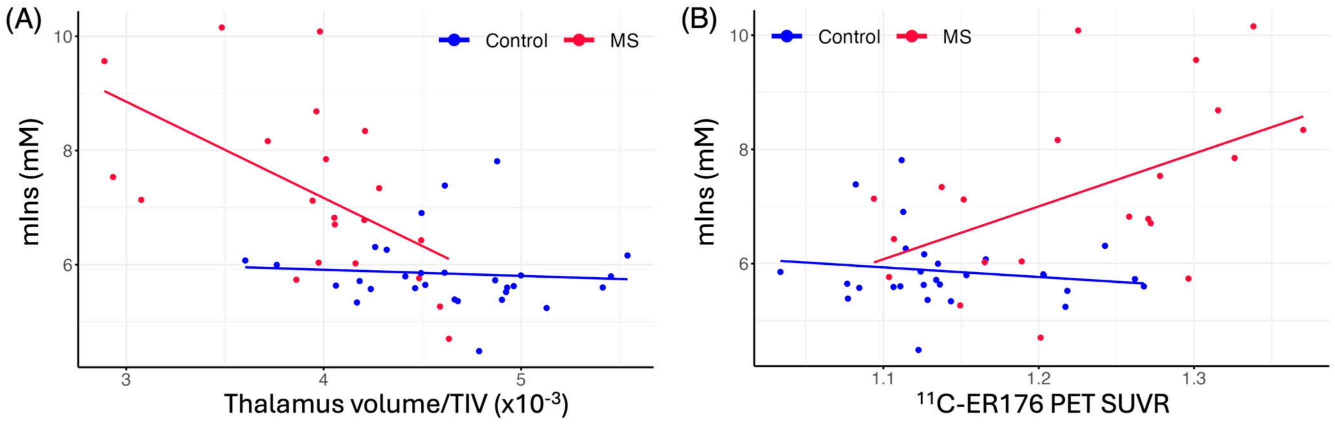


**Figure S1.** Pearson correlation of increased myo-inositol concentration (mIns) (mM) with decreased normalized thalamic volume (A) and increased $^{11}$C-ER176 TSPO PET SUVR (B). The red scatter plots represent data from patients with multiple sclerosis (MS). The blue scatter plots represent data from controls.  These graphs demonstrate correlations without age adjustment. Correlation coefficients (A): control (r= -0.08, p=0.687), pwMS (r=-0.56, p= 0.009); Correlation coefficients (A): control (r= -0.15, p=0.437), pwMS (r=0.52, p= 0.017). Supplementary Tables S5 and S6 demonstrate correlation coefficients of Pearson correlation adjusted for age and the corresponding confidence interval and p-values. Abbrevaitions: myo-inositol; TIV, total intracranial volume.

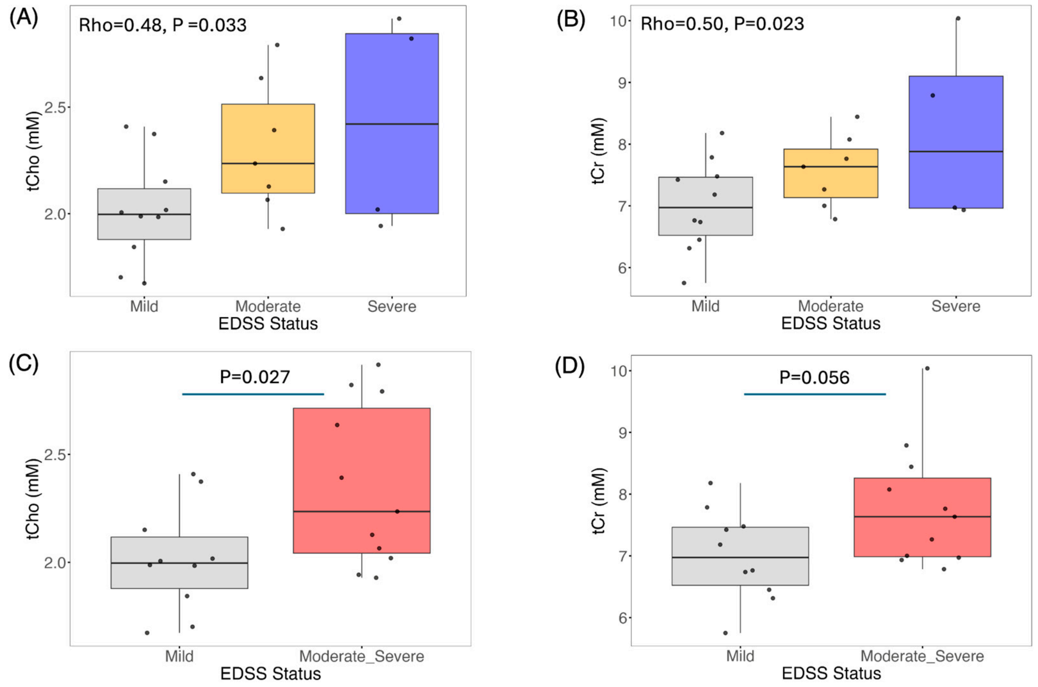


**Figure S2.** The associations of $^{1}$H-MRS metabolites with clinical metrics in patients with multiple sclerosis. Increased total choline (tCho) (A) and increased total creatine (tCr) (B) estimated concentrations (millimolar, mM) were associated with increased disability, as measured by the Expanded Disability Status Scale (EDSS) (mild: Group 1=EDSS 0.0-2.5; moderate: Group 2= EDSS 3.0-5.5; severe: Group 3= EDSS≥6.0). Total choline was significantly increased in the combined EDSS moderate and severe groups compared to the mild group (two-tailed t-test, p = 0.027) (C). However, tCr did not show a statistically significant difference between Mild and combined EDSS group (moderate plus severe) (two-tailed t-est) (p=0.056) (D). Age-adjusted Spearman correlation coefficients (rho) and p-values for the relationship between tCho, tCr and EDSS are shown in the upper-right corner of each figure (A, B). For confidence intervals of partial Spearman correlations adjusted for age, refer to supplementary Table S6. P-values are rounded to three decimal places.
